# Differential Orbitofrontal Cortex Responses to Chocolate Images While Performing an Approach–Avoidance Task in the MRI Environment

**DOI:** 10.3390/nu15010244

**Published:** 2023-01-03

**Authors:** Anja Lender, Janina Wirtz, Martin Kronbichler, Sercan Kahveci, Simone Kühn, Jens Blechert

**Affiliations:** 1 Department of Psychology, University of Salzburg, Hellbrunner Straße 34, 5020 Salzburg, Austria; 2 Centre for Cognitive Neuroscience, University of Salzburg, Hellbrunner Straße 34, 5020 Salzburg, Austria; 3 Department of Psychiatry and Psychotherapy, Neuronal Plasticity Working Group, University Medical Center Hamburg, Eppendorf Martinistrße 52, 20246 Hamburg, Germany; 4 Neuroscience Institute, Christian Doppler Medical Centre, Paracelsus Medical University, 5020 Salzburg, Austria; 5 Lise Meitner Group for Environmental Neuroscience, Max Planck Institute for Human Development, Lentzeallee 94, 14195 Berlin, Germany

**Keywords:** approach bias, AAT, craving, chocolate, fMRI

## Abstract

**Background:** Chocolate is one of the most frequently craved foods, and it often challenges self-regulation. These cravings may be underpinned by a neural facilitation of approach behavior toward chocolate. This preregistered study investigated the behavioral and neural correlates of such a bias using functional magnetic resonance imaging (fMRI) and reaction times (RTs). **Methods:** A total of *n* = 30 frequent chocolate eaters performed a relevant-feature approach–avoidance task (AAT) in the MRI scanner using buttons to enlarge (approach) or to shrink (avoid) pictures of chocolate and inedible control objects. We tested (a) whether implicit RT-based approach biases could be measured in a supine position in the scanner, (b) whether those biases were associated with activity in reward-related brain regions such as the insula, amygdala, striatum, and orbitofrontal cortex (OFC), and (c) whether individual RT-based bias-scores correlated with measures of chocolate craving. **Results:** Behaviorally, we found a highly reliable approach bias toward chocolate, defined by faster RTs in the compatible conditions (approach chocolate, avoid objects) compared to the incompatible conditions (avoid chocolate, approach objects). Neurally, this compatibility effect involved activity in the left medial OFC, a neural response that was positively correlated with individual approach bias scores. **Conclusions:** This study shows that the relevant feature AAT can be implemented in an fMRI setting in a supine position using buttons. An approach bias toward chocolate seems related to medial OFC activation that might serve to devalue chocolate when it has to be avoided. Our demonstration of neural and behavioral approach biases for chocolate underscores the need for stimulus-specific cognitive trainings to support healthy consumption and successful self-regulation.

## 1. Introduction

While consuming food is essential for survival, overconsumption of unhealthy foods can cause a variety of diseases, such as diabetes, cancer, and cardiovascular disease [1,2]. Overeating is often related to food craving, which is an intense desire to consume a specific type of food. Food craving, in turn, is frequently elicited by external food cues [3]. Food craving can be distinguished from hunger, which can be satisfied by any food, while food craving is more intense and directed toward a certain type of food [4]. Food craving is related to food addiction, loss-of-control eating [5,6,7], and unsuccessful dieting [8]. At the same time, it is a common phenomenon in binge eating and related disorders [6,9], as well as in subclinical eating disorders and obesity [3,10,11]. Strong cravings can lead to suffering in affected individuals, caused by the conflict between health- and weight-related goals, on the one hand, and the desire to consume unhealthy foods, on the other hand.

When exposed to craved substances, it can be difficult for individuals to consciously inhibit their automatic consummatory action tendencies [12,13,14]. According to dual-process models, during information processing, there are two systems evaluating the presented stimuli: the ‘bottom-up’ system, which is thought to operate in a quick and effortless manner, and the ‘top-down’ system, which is slower and involves more effort as it is deliberate and reflective [11,15,16]. In addictive behaviors, there appears to be an imbalance between the bottom-up and top-down systems, leading to a relative dominance of the appetitive tendencies invoked by the bottom-up system and, consequentially, a stronger approach tendency toward craved stimuli [15,17,18]. The bottom-up system prepares an individual’s behavior in a relatively automatic fashion, except when the top-down system intervenes to regulate it. Thus, it is of key importance to identify the behavioral and neural correlates of the bottom-up system, as this could inform intervention efforts [19].

Impulsive eaters are thought to have stronger approach biases toward unhealthy food cues [20,21]. An approach bias is the tendency to more readily approach than avoid appetitive stimuli [15], and it can be assessed using the approach–avoidance task (AAT). In the most common version of this computerized task, participants respond to visual stimuli of positive, negative, or neutral valence by either enlarging (approaching) or shrinking (avoiding) the stimulus, e.g., by pulling or pushing a joystick, respectively. The zoom effect symbolizes distance change in naturalistic (horizontal) approach–avoidance movements. The task instructions determine on which trials participants need to approach or avoid the stimulus. In general, responses are performed more quickly when they are compatible with stimulus valence (i.e., approach positive stimuli) rather than incompatible (i.e., avoid positive stimuli) or when they are unrelated to the stimulus valence, such as with neutral stimuli. An approach bias toward high-calorie foods could be demonstrated in food cravers in some studies [11,22,23,24], while other studies could not confirm an approach bias toward high-calorie foods [22,25]. Yet, other studies found approach bias mainly toward individually liked foods [26], low-calorie foods [27], or specifically chocolate [23]. These inconsistent findings might be caused by a state or context dependency of the approach bias toward food cues [28].

Neural processes underlying the performance of the AAT have been explored in a variety of fields, such as social cognition [29], depression [30], and consumption of alcohol [31] and high-calorie foods [32]. Imaging studies in addiction revealed neural differences between alcohol-dependent patients and healthy controls while performing an alcohol AAT; compared to controls, patients had stronger activation in reward-related brain regions (nucleus accumbens and medial prefrontal cortex) while approaching alcohol. In contrast, no differences between patients and controls were found in frontal control regions (dorsolateral prefrontal cortex) while avoiding alcohol. Differences in performance in the alcohol AAT can, thus, better be explained as differences in automatically engaged approach processes rather than in control-induced avoidance processes [31,33]. In contrast, research in obesity has implicated inhibition-related, top-down controlled processes during the performance of a food AAT. Individuals with obesity showed stronger activation in brain regions associated with attentional reorientation and behavioral inhibition (right angular gyrus) while avoiding unhealthy foods. In contrast, no effect was found in brain regions associated with reward processing. Obesity-related differences in performance on the food AAT, thus, seem to implicate systems of controlled avoidance behavior [32]. A meta-analysis of 14 studies [34] demonstrated that exposure to visual high-calorie food cues is related to higher activation in brain areas associated with reward processing, namely, the insula, striatum, amygdala, and orbitofrontal cortex (OFC). Neurocognitive models suggest a strong connection between the motivation to consume certain foods and the brain’s reward system. This connection might mediate the biased processing of these foods, as well as ‘incentive saliency’, attentional biases, and approach biases [35,36,37].

The current study focused on habitual chocolate eaters, who are often unable to control their intake and, according to dual-process models, unable to override the ‘bottom-up’ system. We employed an AAT in the MRI scanner and tested whether a behavioral approach bias would be present and, if so, whether it would be correlated with activation in reward-related brain regions that have been shown to be active during the passive neural processing of food images. To ensure that participants focus their attention on the stimuli and evaluate their content during the performance of the AAT, this study used a relevant feature AAT instruction. Previous food AAT studies [32] required participants to approach or avoid on the basis of content-independent features of the stimulus (vertical vs. horizontal orientation); the present study instead requires participants to approach or avoid depending on the content of the stimulus. Relevant feature AATs have been shown to yield more robust and reliable approach biases than irrelevant feature ones [24,38,39,40,41]. This setup might activate brain regions associated with reward (i.e., nucleus accumbens) as found in alcohol AAT studies [31,33]. Perceived distance change is thought to mediate approach biases; thus, it was of particular interest to see whether reliable behavioral biases could be obtained in the fMRI where images approach and avoid the participant ‘from above’ due to the supine position, which is at odds with naturalistic approach behavior. Interestingly, previous research has rarely reported on absolute approach biases in the scanner, and no previous research has reported reliability indices. To validate the approach bias on a behavioral and neural level, we also estimated its association with state chocolate craving. In line with studies on alcohol-dependent patients [31,33], it was hypothesized that participants with high-state chocolate craving have a stronger behavioral approach bias toward chocolate, which is associated with increased activation in reward-related brain regions (insula, striatum, amygdala, and OFC).

## 2. Methods

Hypotheses and statistics were specified in a preregistration in the Open Science Framework (OSF) [42]. Additional (not preregistered) analyses are declared exploratory.

### 2.1. Participants

We invited participants with a strong preference for chocolate and habitual chocolate consumption. Out of the 195 people who replied to our online announcement, 126 completed our online survey, including the assessment of trait chocolate craving (chocolate version of the Food Cravings Questionnaire—Trait, FCQTr-chocolate [43]) and chocolate consumption (‘How many days per week do you eat chocolate?’). We utilized a range of inclusion and exclusion criteria. Only participants with strong trait chocolate craving (FCQTr-chocolate > 40) and high chocolate consumption (three or more days per week) were invited for further participation. Furthermore, participants had to be right-handed and between 18 and 60 years old. Exclusion criteria were food allergies or a vegan diet, as well as neurological or psychological disorders. For safety reasons, participants with ferromagnetic implants, claustrophobia, or pregnancy were excluded. On the basis of this preselection, 40 participants were invited to our laboratory. Six missed the appointment, three had to abort their participation as they did not feel well in the scanner, and one participant did not follow the task instructions. We stopped our data collection after the 30th full dataset, which was our predefined sample size according to studies with similar tasks [32,44]. In our final sample, 20 (66.7%) participants were female, the average age was 31.9 (SD = 11.6), and the average body mass index (BMI) was 23.5 (SD = 4.02). Participants received a reimbursement of 20 EUR. The study was approved by the ethics committee of the University Medical Center Hamburg-Eppendorf (LPEK-0038), and participants signed an informed consent form before participating.

### 2.2. Stimuli

Stimuli were selected from ‘food-pics_extended’, a freely available database of food images with normative data for the study of eating behavior [45]. The current stimulus set comprised 16 images of chocolate-containing snacks and 16 images of inedible control objects. All images depicted items that are easy to grab. Stimuli were selected to be recognizable and familiar, based on normative data from food-pics (selection threshold: >90 out of 100 points on a visual analogue scale). Chocolate and control images did not differ in these ratings and in image properties, such as color, brightness, contrast, complexity, and spatial frequency. In the present study, participants rated the stimulus set on valence and on palatability (see Appendix A).

### 2.3. AAT

We used a relevant feature version of the AAT, where participants had to ‘approach’ or to ‘avoid’ visual stimuli according to image category (chocolate sweets vs. inedible objects) [24,38,39,40]. To approach a stimulus, participants pressed a lower key on a button-stick, which zoomed the image in. To avoid a stimulus, participants pressed an upper key, which zoomed the image out. Each trial started with a fixation cross (2000–5000 ms), followed by the stimulus, with both presented in the center of the screen. If the participant did not respond within 1500 ms (timing error trial), a warning sign was displayed, requesting the participant to react faster. If the participant did not respond with the correct key (response error trial), the image disappeared without zooming in or out, and the next trial started. If the participant reacted with the correct response in time (correct trial), the image zoom started and was followed by the next trial.

**Figure 1:**
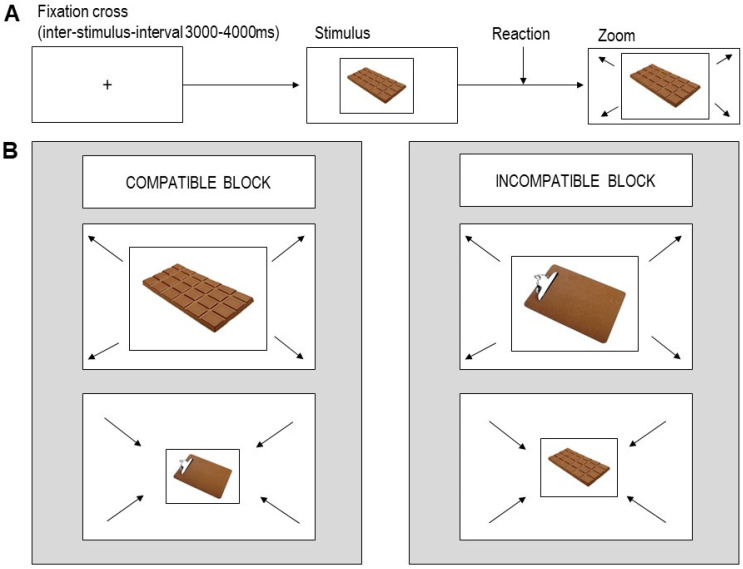
(**A**) Timeline of a single trial. (**B**) Task setup: relevant feature approach–avoidance task.

The task comprised two blocks (see Figure 1). In the ‘compatible’ block, participants were instructed to approach chocolate and avoid objects. In the ‘incompatible’ block, participants were instructed to avoid chocolate and approach objects. The order of the two blocks was counterbalanced across participants. Each block included 64 chocolate image trials, 64 object image trials (four repetitions of each individual image), and 25 null events (1500 ms fixation cross), resulting in 153 trials per block, presented in a pseudo-randomized sequence. The inter-stimulus intervals (ISI) and the sequence of trials were calculated using a Matlab tool for fMRI design optimization [46].

### 2.4. Procedure

To limit how much the data were influenced by circadian patterns of hunger and craving, testing took place between 1 and 6 p.m., and participants were requested to consume a regular meal and then refrain from eating for 3 h before the experiment. Furthermore, to avoid satiation for sweets, participants were requested not to eat any sweets during the day of the scan.

Participants signed the consent form, reported sociodemographic data, and completed the chocolate version of the Food Cravings Questionnaire—State (FCQS-chocolate [43]), to assess the current state of hunger and chocolate craving. A bowl of sweets was presented to participants before they went into the MRI scanner, as a previous study indicated that the immediate availability of palatable foods increases palatability ratings and reward-related neural responses toward images of these foods [47]. It was mentioned that these sweets resemble the experimental stimuli and would be available for takeaway after the experiment. Participants were then offered a small piece of chocolate to be eaten immediately before scanning. This kind of preload has been shown to increase palatability as well as the consumption of sweet items [48]. In the scanner, participants first completed a cue reactivity task during which they passively observed the 32 stimuli used in the AAT (data not presented here). This task was followed by the AAT. After instructions and practice trials with food-unrelated stimuli, participants completed the AAT. After scanning, participants received remuneration and were offered the sweets they had seen before. The number of sweets taken home was documented as a naturalistic measure of approach motivation.

### 2.5. Behavioral Data Analysis

To determine the approach bias toward chocolate, the median RT was calculated across correct trials within each condition (approach chocolate, avoid chocolate, approach objects, and avoid objects). Incorrect trials with RT above 1500 ms or below 200 ms, as well as incorrect trials with wrong button presses, were excluded. Following 64 trials within each condition, *M* = 2.43 (*SD* = 2.01) incorrect trials had to be removed in the approach chocolate condition, in contrast to *M* = 3.33 (*SD* = 3.59) in the avoid chocolate condition, M = 4.87 (*SD* = 2.73) in the approach objects condition, and *M* = 3.60 (*SD* = 3.16) in the avoid objects condition. The criterion of ≥41 correct trials (65%) [49] within each condition was fulfilled by all participants; thus, none had to be excluded due to an insufficient number of valid trials. Median RTs within the four conditions were submitted to a repeated-measures analysis of variance (ANOVA) with stimulus type (chocolate vs. objects) and direction (approach vs. avoid) as within-participant factors. A general approach bias was defined as a significant interaction, indicating a speeded approach of chocolate relative to all other conditions (*p* < 0.05), confirmed by post hoc *t*-tests (*p* < 0.05, Bonferroni-corrected).

As preregistered, validity of the approach bias was tested by the convergence between individual approach bias scores and subjective experience of state chocolate craving. Individual approach bias scores were calculated by double-difference scores of median RTs in the four conditions: (RT_avoid chocolate_ − RT_approach chocolate_) − (RT_avoid objects_ − RT_approach objects_). The subjective experience of state chocolate craving was represented by the total score of the FCQS-chocolate. Reasonable convergent validity was defined as a significant correlation between individual approach bias scores and total scores of the FCQS-chocolate (*p* < 0.05).

The permutated split-half reliability of the approach bias scores was computed using a package (‘AATtools’ [50]) for R [51]. In this tool, participants’ correct trials were randomly split into two halves, with an almost equal number of trials of each condition and participants in each half (approach chocolate, avoid chocolate, approach objects, and avoid objects). Next, approach bias scores were calculated for the two subsets separately and correlated with each other across participants. This procedure was performed 10,000 times. The split-half reliabilities across all permutations (with 95% confidence interval) were averaged and then Spearman–Brown-corrected to account for halved datasets. As preregistered, a robust reliability was defined by a Spearman–Brown corrected correlation above 0.60.

### 2.6. MRI Data Analysis

Neural data were acquired on a 3 T MRI scanner (Siemens Prisma) equipped with a 64-channel head coil. A T2*-weighted echo planar imaging (EPI) sequence was used to acquire functional images with blood oxygenation level (BOLD) contrast (repetition time = 2000 ms, echo time = 30 ms, image matrix = 64 × 64, field of view = 216 mm, flip angle = 80°, slice thickness = 3.0 mm, distance factor = 20%, voxel size = 3 × 3 × 3 mm^3^, 36 axial slices, using GRAPPA). A T1-weighted magnetization-prepared rapid-acquisition gradient echo (MPRAGE) sequence was used to acquire structural images for co-registration (repetition time = 2500 ms, echo time = 2.12 ms, TI = 1100 ms, acquisition matrix = 240 × 241 × 194, flip angle = 9°, 0.8 × 0.8 × 0.94 mm voxel size). All preprocessing steps and subsequent analysis were performed in SPM12 [52]. The first five volumes of each functional run were discarded to allow the magnetization to stabilize to a steady state. During preprocessing, functional images were spatially realigned, slice-time-corrected to the onset of the first slice, co-registered to the high-resolution structural images, normalized to Montreal Neurological Institute (MNI) space, and finally smoothed with a Gaussian kernel of 8 mm full width at half maximum.

On the participant-specific first level, stimulus onsets (represented by stick functions) were convolved with a canonical hemodynamic response function and its first temporal derivative. Each of the four conditions (approach chocolate, avoid chocolate, approach objects, and avoid objects) was represented by one regressor in the participant-specific general linear model. In addition, incorrect trials were modeled as a nuisance regressor (correct vs. incorrect) to avoid error related brain activity in the implicit baseline. Hence, the implicit baseline included almost exclusively null events. Furthermore, 12 realignment parameters were included as nuisance regressors to minimize residual variance. The first six nuisance regressors corrected for head movements (three directions of translation, three axes of rotation). The last six nuisance regressors corrected for non-neural high-temporal-amplitude changes and temporal instability in spatial brain regions, identified by FIACH (functional image artefact correction heuristic), a procedure that has been proven to increase the robustness of fMRI data in the context of task-induced movement [53]. Finally, the data were high-pass filtered with a cutoff of 128 s. On the basis of this general linear model, parameter estimates for each condition were calculated and used to build participant-specific contrasts representing brain activation related to an approach bias toward chocolate (compatible > incompatible; compatible < incompatible). This contrast is in accordance with the behavioral double-difference score (the approach bias score), indicating a difference between compatible (approach chocolate and avoid objects) and incompatible conditions (approach objects and avoid chocolate).

On the group-related second level, the participant-specific contrasts were entered into one-sample *t*-tests (*p* < 0.05 FWE-corrected, *k* > 5). For a priori defined (preregistered) regions of interest (striatum, amygdala, insula, and OFC), AAL masks of the WFU PickAtlas were used for small volume correction (SVC) (*p* < 0.05, FWE-corrected at cluster level). For exploratory purposes, approach bias-related brain activation in regions of interest was validated with behavioral measures. Therefore, beta-estimates within preregistered AAL masks were extracted using MarsBaR [54] and correlated with individual approach bias-scores (*p* < 0.05).

## 3. Results

### 3.1. Behavioral Results

**Figure 2:**
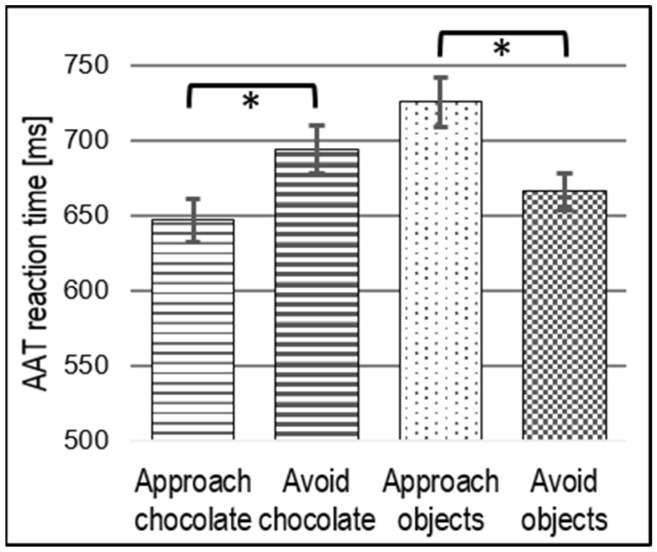
RTs for each AAT condition indicating a general approach bias toward chocolate; * *p* < 0.05.

RT measures during the AAT revealed a stimulus main effect (*F_(1, 29)_* = 17.60, *p* < 0.001, *η^2^_p_* = 0.37) that was modulated by a direction (approach vs. avoid) × stimulus type (chocolate vs. objects) interaction with large effect size (*F_(1, 29)_* = 9.81, *p* = 0.004, *η^2^_p_* = 0.25). This indicates faster responses in the compatible conditions (approach chocolate and avoid objects) than in the incompatible conditions (avoid chocolate and approach objects), as shown in Figure 2. The post hoc *t*-test revealed faster reactions (*t*_(29)_ = 2.57, *p* = 0.020, Bonferroni-corrected) in approaching compared to avoiding chocolate, but slower RTs (*t*_(29)_ = 3.26, *p* = 0.002, Bonferroni-corrected) in approaching compared to avoiding objects.

A post hoc power analysis was performed by sampling participants with replacement from our dataset and performing the same stimulus × direction ANOVA predicting median RT. Sample sizes were tested from five to 50 in steps of five; for each sample size, we repeated the aforementioned sampling and analysis process 200 times. The post hoc power was obtained by computing the proportion of samples at each sample size in which *p* was below 0.05 for the interaction of movement direction and stimulus category. At our sample size of 30 participants, we had sufficient power to detect the effect, we observed in the previous paragraph (1 − β = 0.88); an acceptable power above 0.80 was already achieved at a sample size of 25 (1 − β = 0.84).

As other research has shown order effects [26,55], we explored whether order effects were present in the current data. Adding order as a between-subject factor to the ANOVA revealed a significant three-way interaction, indicating that individual approach bias scores depended on AAT block order (*F_(1, 28)_* = 18.60, *p* < 0.001, *η^2^_p_* = 0.39). Post hoc ANOVAs in each block order condition revealed that approach bias was highly significant in participants completing the compatible block first (*F_(1, 14)_* = 29.30, *p* < 0.001, *η^2^_p_* = 0.59), but failed to reach significance in participants completing the compatible block second (*F_(1, 14)_* = 0.07, *p* < 0.795, *η^2^_p_* = 0.001). In those completing the compatible block first, post hoc *t*-tests revealed faster RTs (*t*_(14)_ = 5.05, *p* = 0.001) in approaching (*M* = 630 ms, *SD* = 78 ms) compared to avoiding chocolate (*M* = 737 ms, *SD* = 92 ms), but slower RTs (*t*_(14)_ = 5.42, *p* = 0.001) in approaching (*M* = 752 ms, *SD* = 103 ms) compared to avoiding objects (*M* = 636 ms, *SD* = 62 ms). When completing the compatible block second, post hoc *t*-tests revealed no difference in RTs (*t*_(14)_ = 0.627, *p* = 1) between approaching (*M* = 663 ms, *SD* = 78 ms) and avoiding chocolate (*M* = 650 ms, *SD* = 57 ms), as well as no difference in RTs (*t*_(14)_ = 0.195, *p* = 1) between approaching (*M* = 698 ms, *SD* = 67 ms) and avoiding objects (*M* = 694 ms, *SD* = 61 ms).

Independent of block order, there was a significant difference in RT between blocks (*t*_(29)_ = 3.50, *p* = 0.001), such that participants became slower from the first block (*M* = 654 ms, *SD* = 67 ms) to the second block (*M* = 712 ms, *SD* = 86 ms).

Validity of the approach bias could not be demonstrated by a significant correlation between individual approach bias scores and state chocolate craving or other measures from several questionnaires, stimulus ratings, or chocolate items taken back home (see Appendix B).

The permutated split-half procedure (with 10,000 iterations) confirmed a robust split-half reliability of the approach bias scores (Spearman–Brown coefficient): SB_(28)_ = 0.95, 95% CI [91, 97].

### 3.2. MRI Results

**Figure 3:**
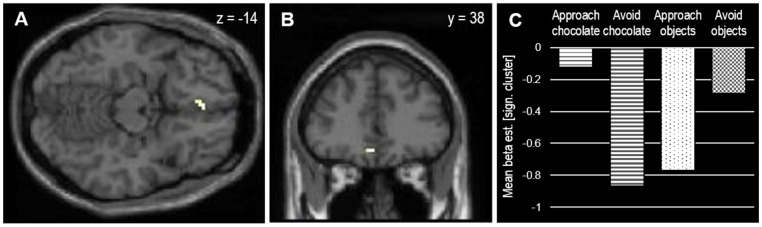
(**A**,**B**) Significant cluster in left medial OFC associated with approach bias toward chocolate (*p* = 0.001 uncorrected at whole brain level, *k* > 5). (**C**) Extracted beta-estimates from significant cluster indicating stronger deactivation (relative to baseline and null events) under incompatible conditions (avoid chocolate and approach objects) compared to compatible conditions (approach chocolate and avoid objects).

The next analysis was an MRI contrast reflecting an approach bias (compatible > incompatible), calculated by the difference between compatible (approach chocolate and avoid objects) and incompatible conditions (avoid chocolate and approach objects). It revealed no effects using the preregistered conservative statistical threshold (*p* = 0.05, FWE-corrected at whole brain level, *k* > 10). However, by using a more liberal threshold (*p* = 0.001 uncorrected at whole brain level, *k* > 5), brain activation was stronger in the compatible (approach chocolate and avoid objects) than in the incompatible conditions (avoid chocolate and approach objects) in two clusters in the right medial occipitotemporal cortex and the left medial OFC (Figure 3, Table 1).

As the OFC was a preregistered region of interest, SVC was conducted using an AAL mask for left medial OFC. This approach confirmed the contribution of the medial OFC to the approach bias in the AAT (*p_svc_* = 0.023 FWE-corrected at the cluster level).

**Figure 4:**
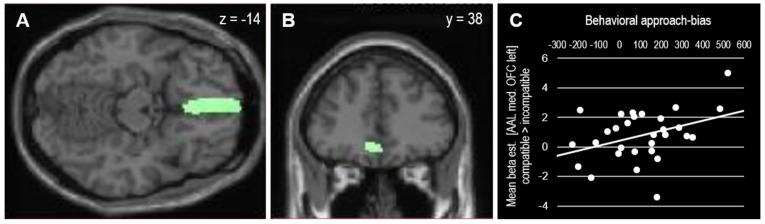
(**A**,**B**) Mask (AAL medial OFC left) used for SVC (*p*_svc_ = 0.023 FWE-corrected at cluster level). (**C**) Extracted beta-estimates from OFC mask and their association with behavioral approach bias scores (*r*_(28)_ = 0.381, *p* = 0.038).

This approach bias-related OFC activation correlated positively with the behavioral approach bias scores (*r*_(28)_ = 0.38, *p* = 0.038), as depicted in Figure 4. To take the block-order effect into account, the correlation was computed for both orders separately. In line with the pattern observed in the behavioral analysis, the correlation between individual approach bias scores and medial OFC activation was mainly driven by participants who performed the compatible block at first (*r*_(13)_ = 0.72, *p* = 0.003) but not by participants who performed the compatible block as the second block (*r*_(13)_ = −0.07, *p* = 0.807).

The opposite contrast (incompatible > compatible) revealed lower brain activation in the compatible (approach chocolate and avoid objects) than in the incompatible conditions (avoid chocolate and approach objects) in the left caudate (*p* = 0.001 uncorrected at whole brain level, *k* > 5), as depicted in Table 1. As the caudate (striatum) was a preregistered region of interest, SVC was conducted using an AAL mask for the left caudate. However, the cluster only reached trend-level significance (*p_svc_* = 0.068 FWE-corrected at cluster-level). For MRI contrasts from the whole-brain analysis regarding the stimulus type (approach chocolate and avoid chocolate vs. approach objects and avoid objects) please see Appendix C.

## 4. Discussion

The present study investigated neural activation patterns during approach behavior toward chocolate in the MRI scanner. As perceived distance change is thought to mediate approach bias, it was of particular interest to see whether reliable behavioral biases could be obtained in the fMRI customary supine position, which is at odds with naturalistic approach behavior performed in upright position. Interestingly, previous research has rarely reported on absolute approach biases toward appetitive stimuli in the scanner, and it has not reported reliability indices [31,32,33]. The study had two aims. The first aim was to assess whether an approach bias in habitual chocolate eaters has a distinct neural signature. The second aim was to assess whether inter-individual differences in this approach bias are reflected in stronger or weaker activation in food- and reward-related brain regions, such as the insula, amygdala, striatum, and OFC [32]. Individual approach bias scores were assessed with an MRI-suitable AAT, using buttons to enlarge (approach) or to shrink (avoid) chocolate items or inedible control objects. A main finding on the behavioral level was a general approach bias toward chocolate, defined as faster responses in the compatible conditions (approaching chocolate and avoiding objects) compared to the incompatible conditions (avoiding chocolate and approaching objects). This behavioral effect was accompanied by a relatively stronger activation in the left medial OFC in the compatible conditions compared to the incompatible conditions. Supporting its potential role in biasing behavioral approach responses, the left medial OFC activation was positively related to the behavioral approach bias scores. In contrast to our expectations, the approach bias scores did not correlate with state chocolate craving. Likewise, several regions of the reward system did not show compatibility effects in contrast to our hypotheses.

The medial OFC is considered an early key structure in the processing of reward value. It relays reward value information to the anterior cingulate cortex, where it is used for action–outcome learning [56]. Imaging studies on eating behavior have shown that the individual reward value, measured by pleasantness ratings, correlates positively with medial OFC activation, and that this association is more pronounced in food cravers [57,58,59]. Furthermore, it has been found that both pleasantness ratings and medial OFC activation were higher during hunger than during satiation when researchers modulated individual reward value with hunger manipulations [58,60]. Interestingly, hunger manipulation was found to interact with energy content, such that hunger increased medial OFC activation particularly for high-calorie foods, and energy content correlated positively with medial OFC activation when foods were evaluated [60]. Furthermore, decreased medial OFC activation covaried with sensory-specific satiety effects. In one study, participants consumed one of two drinks (chocolate milk vs. tomato juice) until they were satiated. Subsequently in the scanner, the OFC was less activated by images of the drink that were previously consumed [58]. Lastly, a stronger food-cue induced activation of the medial OFC predicted subsequent choice of foods with higher fat content [61]. From this perspective, the current OFC effect may represent differences in reward-value processing in the compatible and incompatible conditions in the AAT.

The current results complement previous findings from AAT studies investigating underlying neural processes. AAT studies in the alcohol domain have linked performance in the AAT to bottom-up processes driving automatic approach behavior, represented by activation in reward-related brain regions (nucleus accumbens and medial prefrontal cortex) [31,33]. In contrast, an AAT study in the food context has linked performance on the AAT to top-town processes driving controlled avoidance behavior, represented by activation in brain regions associated with attentional reorientation and behavioral inhibition (right angular gyrus) [32]. In line with this latter finding, we also did not find an involvement of preregistered reward-related structures (striatum, amygdala, and insula). Thus, food stimuli may facilitate the approach bias through different processes than alcohol stimuli. Pharmacologically active substances might differ from food in their incentive salience, due to their direct physiological effects on the brain following consumption. While alcohol stimuli increase incentive salience to trigger automatic approach tendencies via dopaminergic fronto-limbic reward structures [62,63], food stimuli might not trigger the reward system in the same way [64].

The only preregistered reward-related region that showed a significant a compatibility effect was the medial OFC. The contrast between compatible and incompatible conditions in this region can be interpreted in both directions. On the one hand, stronger OFC activation during compatible conditions may indicate a reliance on reward processing of chocolate items to facilitate fast and intuitive responses to familiar action–outcome contingencies. On the other hand, weaker OFC activation during incompatible conditions may represent a downregulation of reward processing to gain better control over responses while learning new action–outcome contingencies [65]. The latter interpretation seems to be more plausible, as the medial OFC contrast appears to be driven by the incompatible conditions (see Figure 2). Furthermore, the interpretation of downregulated reward valuation under incompatible conditions is in line with the theory of behavior stimulus integration (BSI) [65]. BSI proposes an interaction between behavior and stimuli when an approach reaction toward a positive stimulus is unwanted as it slows task performance. To solve this response conflict and associated performance decrements, the stimulus will be evaluated as less positive to alleviate the automatic approach tendency [65]. BSI has been corroborated with different types of appetitive stimuli including foods [66], and the devaluation effect was stronger when attentional focus had to be directed to the stimulus [67]. According to dual-process models [11,15,16], the current medial OFC effect may, thus, represent top-down control over reward processing while avoiding chocolate items.

The current findings have implications for the implementation of food AAT interventions. In these cognitive bias modification trainings, high-calorie food items are almost always (e.g., 90%) paired with avoidance responses, whereas control stimuli (e.g., neutral items or low-calorie food items) are almost always paired with approach responses. AAT interventions have the potential to help treat food cravings and overweight, as they can affect food choice and food consumption when the control stimuli comprise healthy food items [68]. On the neural level, Mehl et al. (2018) [32] showed that an approach bias modification intervention affected neural activation in brain regions associated with attentional reorientation and behavioral inhibition (right angular gyrus), and it reduced behavioral approach tendencies toward unhealthy food. However, the intervention did not affect brain regions associated with reward or stimulus evaluation, and it did not change the liking of the trained food items. The contrast with the current findings may be explained by differences in experimental design. Mehl et al. (2018) [32] used a task design with an irrelevant feature instruction, where participants had to respond to the format of the picture (horizontal vs. vertical), while the current study used a task design with a relevant feature instruction, where participants had to respond to the content of the picture (chocolate vs. objects). This direct attentional focus on the content may have driven neural processes of reward evaluation, as shown by medial OFC activation. Future research should investigate whether AAT interventions can be improved by a relevant feature instruction, to make AAT interventions comparable to other established stimulus devaluation trainings such as go/no-go trainings [69].

### Limitations and Future Studies

In the current study, statistical power may have been reduced by additional variance due to block-order effects, a common confound in AAT studies using relevant feature instructions [26,55]. In these studies, the order effect is represented by faster reactions in the second block, which may be caused by learning over time. However, in the current study, the order effect is instead represented by *slower* reactions in the second block, which may be caused by increasing fatigue over time. This fatigue may have been caused by the very long duration of the fixation cross preceding the stimuli, which was necessary for the reliable measurement of changes in the BOLD signal. Because the approach bias is based on faster reactions during compatible conditions, the gradually increasing fatigue artificially strengthens the approach bias effect when the compatible block comes first, and it artificially weakens the approach bias effect when the compatible block comes second. We used a strict counterbalancing technique to deal with expected order effects on the group level, but individuals’ bias scores remained affected. For future studies, adapting the experimental design is recommended. One solution to prevent order effects could be an adjustment of the common two-block design to a sequence of multiple alternating blocks [70,71]. More than four blocks may be required; van Alebeek et al. (2021) [71] used a sequence of six blocks and found no order effects, while Kahveci et al. (2021) [26] used four blocks and did find order effects. In general, order effects limit the power to test for correlational validity, a drawback that explains the present failure to find expected correlations between individual approach bias scores and other food-related variables.

Surprisingly, most predefined reward-related brain regions (amygdala, insula, and striatum) did not show detectable compatibility effects, despite the fact that we took several measures to maximize chocolate craving, including the preselection of high chocolate cravers and the induction of chocolate craving using a preload and cue exposure. Nevertheless, one predefined reward-related brain region, the medial OFC, showed a compatibility effect. This effect was small and variable, and it precluded a detailed model of how behavioral biases are neurally implemented, but its correlation with the behavioral data supports the potential role it may play in the generation of an approach bias toward chocolate. Further research is needed to identify the relevance of the medial OFC-related compatibility effect in the clinical context. It would be of interest to learn whether an AAT intervention can influence medial OFC responses, and whether these medial OFC responses relate to stimulus devaluation. Additionally, neural compatibility effects may be restricted to a subset of trials (e.g., the beginning of each block) or evolve dynamically, masking other effects in the present analysis. Single-trial studies or connectivity studies could address this further, ideally in the absence of order effects that limited the power of the current study.

## 5. Conclusions

The current study investigated the neural processes underlying the performance of symbolic approach and avoidance movements toward chocolate and objects in an AAT that required responses to the content of the picture. We measured a reliable behavioral approach bias toward chocolate, which moderately correlated with a decrease in left medial OFC activation when participants had to avoid chocolate or approach objects, indicating that the approach bias effect may be driven by individual differences in the degree to which reward devaluation is engaged when avoiding desired foods. However, this compatibility effect in the medial OFC was quite small, and the underlying cognitive processes are still unclear. Hence, further research is needed to verify the importance of the medial OFC in stimulus devaluation trainings.

## Figures and Tables

**Table 1:** Results from whole brain analysis for approach bias-related brain activation (statistical threshold: *p* < 0.001 uncorrected, *k* > 5 voxels).

Contrast	Brain Area	Voxels	MNI [x, y, z]	*T*_max_
Compatible > incompatible				
	R medial occipitotemporal	6	24, −76, −2	3.53
	L medial orbitofrontal	7	−6, 38, −14	3.42
Incompatible > compatible				
	L caudate nucleus	7	−6, −17, 7	4.05

Labels of brain areas are taken from SPM AAL masks.

## Data Availability

The data and syntax (including a documentation of all analyses that were undertaken) are available upon request.

## Trial Registration

The trial was preregistered on the Open Science Framework (OSF) (https://osf.io/ysbrp) on 9 December 2019 prior to any analyses being undertaken.

## Appendix A. Comparison of Chocolate vs. Control Items

**Chocolate**	**pic: 0137**	**pic: 0107**	**pic: 0163**	**pic: 0465**	**pic: 0286**	**pic: 0173**	**pic: 0289**	**pic: 0189**
	**pic: 0004**	**pic: 0166**	**pic: 0140**	**pic: 0165**	**pic: 0111**	**pic: 0510**	**pic: 0168**	**pic: 0169**
**Control**	**pic: 1188**	**pic: 1015**	**pic: 1095**	**pic: 1045**	**pic: 1146**	**pic: 1260**	**pic: 1250**	**pic: 1196**
	**pic: 1265**	**pic: 1031**	**pic: 1268**	**pic: 1047**	**pic: 1270**	**pic: 1004**	**pic: 1279**	**pic: 1055**

	**Chocolate**		**Control**		**Statistics**	
	***n* = 16**		***n* = 16**			
	*M*	*SD*	*M*	*SD*	*t*_(29)_	*p*
Valence ^1^	5.8	0.23	2.83	0.77	14.7	0.001
Valence ^2^	53.29	6.01	47.63	7.22	2.4	0.023
Recognizability ^2^	96.53	3.72	97.46	3.21	−0.76	0.455
Familiarity ^2^	97.01	2.96	98.13	2.31	−1.19	0.242
Red ^3^	0.47	0.02	0.46	0.06	0.81	0.441
Green ^3^	0.31	0.02	0.31	0.03	−1.20	0.241
Blue ^3^	0.22	0.02	0.23	0.05	−0.26	0.798
Size ^3^	0.31	0.11	0.33	0.12	−0.62	0.542
Brightness ^3^	45.91	20.77	43.52	17.67	0.35	0.728
Contras t^3^	54.24	7.9	46.92	12.79	1.95	0.061
Complexity ^3^	0.07	0.03	0.06	0.03	1.14	0.264
^1^ Rating data collected from the study sample on valence using a seven-point rating scale from ‘not at all pleasant’ to ‘very pleasant’. ^2^ Rating data taken from the Food-Pics data base [45], using visual analogue scales from 0 to 100 h to collect data on valence (‘very negative’ to ‘very positive’), recognizability (‘no’ to ‘yes’), and familiarity (‘no’ to ‘yes’). ^3^ Data on image properties taken from the Food-Pics database [45].						

## Appendix B. Correlations with Approach Bias-Scores

	**All** **(*n* = 30)**		**Compatible Block First (*n* = 15)**		**Incompatible Block First** **(*n* = 15)**	
	***r*_(28)_**	***p***	***r*_(13)_**	***p***	***r*_(13)_**	***p***
State chocolate craving (FCQS)	−0.17	0.357	−0.10	0.722	0.16	0.558
Trait chocolate craving (FCQT)	−0.09	0.629	−0.15	0.589	0.03	0.921
Body-shape concerns (PSRS)	−0.20	0.295	0.06	0.832	−0.20	0.480
Approach motivation (BAS)	−0.11	0.581	0.21	0.452	−0.06	0.839
Chocolate items taken home	0.08	0.698	0.02	0.958	0.06	0.852
Valence rating (chocolate)	−0.22	0.239	−0.03	0.921	0.12	0.671
Palatability rating (chocolate)	−0.19	0.303	0.01	0.965	−0.01	0.974
Trait chocolate craving (chocolate version of the Food Cravings Questionnaire trait, FCQTr—chocolate*1), dieting and body-shape concerns (German version of the Perceived Self−Regulatory Success in Dieting Scale, PSRS [Meule]), approach motivation (German version of the Behavioral Approach System Scale, BAS−scale [Strobel]), and stimulus ratings were assessed during online survey. State chocolate craving (chocolate version of the Food Cravings Questionnaire state, FCQS—chocolate*1) was assessed right before scanning.						

## Appendix C. Results from Whole-Brain Analysis for Stimulus Type

MRI contrasts regarding stimulus type (approach chocolate and avoid chocolate vs. approach objects and avoid objects) revealed a stronger activation by chocolate images compared to control stimuli in the medial occipitotemporal cortex, as well as in the cingulum and the hippocampus (*p* < 0.05 FWE-corrected at whole brain level, *k* > 10). The opposite contrast revealed higher activation by control images compared to chocolate stimuli at several distributed sites. Even though there was no effect in a priori defined (preregistered) regions of interest (striatum, amygdala, insula, and OFC), the current hippocampal effect may relate to the perception of food stimuli, as it has previously been shown that the hippocampus is involved in the regulation of food intake [72].

**Contrast**	**Brain Area**	**Voxels**	**MNI [x, y, z]**	***T*_max_**
chocolate > objects				
	R L medial occipitotemporal	1514	−12, −88, −8	inf
	R hippocampus	12	21, −28, −2	6.19
	L hippocampus	13	−21, −28, −2	6.17
	L middle cingulum	11	−9, −19, 43	5.69
objects > chocolate				
	R cuneus	421	9, −73, 28	6.96
	L superior temporal	15	−45, −31, 7	6.47
	R inferior parietal	81	36, −55, 40	6.24
	L cerebellum	31	−12, −58, −14	5.64
	L postcentral	28	−18, −31, 67	5.62
	L inferior parietal	22	−36, −43, 34	5.34
	R medial temporal	19	54, −26, −11	5.31
	R superior frontal	14	24, 20, 52	5.09
	R superior motor	12	0, −10, 67	5.08
	L angular	12	−33, −58, 34	5.04
Results from whole-brain analysis for stimulus type (statistical threshold: *p* < 0.05 few-corrected, *k* > 10 voxels). Labels of brain areas are taken from SPM AAL masks.				
